# Genome-scale analysis of the high-efficient protein secretion system of *Aspergillus oryzae*

**DOI:** 10.1186/1752-0509-8-73

**Published:** 2014-06-24

**Authors:** Lifang Liu, Amir Feizi, Tobias Österlund, Carsten Hjort, Jens Nielsen

**Affiliations:** 1 Novo Nordisk Foundation Center for Biosustainability, Department of Chemical and Biologicl Engineering, Chalmers University of Technology, SE-41296 Göteborg, Sweden; 2 Novo Nordisk Foundation Center for Biosustainability, Technical University of Denmark, Fremtidsvej 3, DK-2970 Hørsholm, Denmark; 3 Novozymes A/S, Krogshoejvej 36, 2880 Bagsvaerd, Denmark

**Keywords:** *Aspergillus oryzae*, α-amylase, Comparative genomics, Secretory pathway, Transcriptome

## Abstract

**Background:**

The koji mold, *Aspergillus oryzae* is widely used for the production of industrial enzymes due to its particularly high protein secretion capacity and ability to perform post-translational modifications. However, systemic analysis of its secretion system is lacking, generally due to the poorly annotated proteome.

**Results:**

Here we defined a functional protein secretory component list of *A. oryzae* using a previously reported secretory model of *S. cerevisiae* as scaffold. Additional secretory components were obtained by blast search with the functional components reported in other closely related fungal species such as *Aspergillus nidulans* and *Aspergillus niger*. To evaluate the defined component list, we performed transcriptome analysis on three α-amylase over-producing strains with varying levels of secretion capacities. Specifically, secretory components involved in the ER-associated processes (including components involved in the regulation of transport between ER and Golgi) were significantly up-regulated, with many of them never been identified for *A. oryzae* before. Furthermore, we defined a complete list of the putative *A. oryzae* secretome and monitored how it was affected by overproducing amylase.

**Conclusion:**

In combination with the transcriptome data, the most complete secretory component list and the putative secretome, we improved the systemic understanding of the secretory machinery of *A. oryzae* in response to high levels of protein secretion. The roles of many newly predicted secretory components were experimentally validated and the enriched component list provides a better platform for driving more mechanistic studies of the protein secretory pathway in this industrially important fungus.

## Background

*Aspergilli* represents a very important group of cell factories in industrial biotechnology, in particular for the production of industrial enzymes since their high capacity for efficiently secreting both homologous and heterologous proteins allows for cost-competitive production [1]. Compared to other microbial cell factories such as *Escherichia coli* and *Saccharomyces cerevisiae*, *Aspergilli* has a far more complex post-translational modification (PTM) system, which is usually regarded as the bottleneck for protein secretion [2,3]. One of the most widely used *Aspergilli* is the koji mold *A. oryzae*, which due to its long history in soy and rice-based food productions easily obtained the GRAS status [2,4,5]. The fungus produces various industrial enzymes, including amylases, proteases, phytases and lipases *etc.*, representing a market value exceeding 500 million USD. Compared to its extensive industrial applications little is known about its protein secretory machinery. With the whole genome being sequenced in 2005, it has become possible to investigate the protein secretory machinery of *A. oryzae* at the molecular level [6]. However, hindered by its physiological characteristics, e.g. being multinucleate and lacking a sexual life cycle, experimental manipulations on *A. oryzae* is rather difficult compared to other simpler microorganisms, and therefore many of the open reading frames (ORFs) in the genome are still described as hypothetical genes of unknown functions. According to the statistics from the *Aspergillus* Genome Database (AspGD) [7], only 199 of the total 11,703 predicted ORFs have been experimentally verified as of April 24, 2014.

Protein secretion is one of the most complex and important processes in eukaryotes which carries out two main tasks: i) performing proper folding and post translational modifications (PTMs) such as glycosylation and sulfation and ii) sorting cargo proteins to their functional states and final cellular localizations. Secretory components are the proteins handling different processes along the secretory pathway. Recently Feizi *et al.* has constructed a genome-scale model for the protein secretory machinery in *S. cerevisiae*, a model fungus to study many cellular functions including protein secretion [8]. 163 core components involved in the yeast secretory machinery were identified and classified into 16 subsystems based on the processes they involve [8]. The subsystems include Translocation, Dolichol biosynthesis, ER (endoplasmic reticulum) glycosylation, folding, GPI (Glycosylphosphatidylinositol) biosynthesis, GPI transfer, ERADC (ER associated degradation, cytosol), ERADL (luman), ERADM (membrane), COP II (Coat protein complex II), COPI, Golgi processing, LDSV (low density secretory vesicle), HDSV (high density secretory vesicle), CPY (carboxypeptidase Y) pathway and ALP (alkaline phosphatase) pathway [8].

Here we define the functional protein secretory component list of *A. oryzae* using the secretory model of *S. cerevisiae* as a scaffold. The list was further adapted to filamentous fungi by adding *A. oryzae* orthologs of the secretory components reported in other *Aspergillus* species such as *A. nidulans* and *A. niger*. Since amylase production shares resources with other proteins that also perform PTMs on the secretory pathway, in addition to monitor how the secretory components response, we also checked how the fungal secretome was altered in response to amylase overproduction. This analysis has not only provided experimental evidence for the identified secretory components, but also enabled us to understand the secretory machinery in response to high-level protein secretion.

## Results and discussion

### Identification of *A. oryzae* secretory components

Comparing with *A. oryzae*, the genome and the secretory machinery of *S. cerevisiae* is better characterized, and therefore, although less complex, we could still, make use of the yeast secretory pathway to identify and construct an analogous protein secretory pathway of *A. oryzae* using a comparative genomics approach. Through homology search (inparanoid and best hits in AspGD), 121 *A. oryzae* ORFs were mapped to the yeast secretory components with identify over 80% (at the protein level). 37 diverged potential homologs were found through iterative PSI-blast search with significant E-values <0.05 [9]. To complement and accomplish the *A. oryzae* component list, we also included the machinery components previously reported in *A. oryzae*[10-12] and blasted for the *A. oryzae* homologs of the reported components in *A. nidulans*[13] and *A. niger*[11,14], as being in the same genus they share many common mechanisms and pathways. As a result, 83 *A. oryzae* secretory components by Wang *et al.*[10], 5 *A. oryzae* components by Kuratsu *et al.*[12] and 125 components based on blast search (using inparanoid and best hits in AspGD [7]) with the *A. niger* components reported in Oliveira *et al.*[11] were added. Components appeared redundantly in different resources were excluded, and hereby a total of 369 *A. oryzae* genes (putative and experimentally verified) were included in our *A. oryzae* secretory component list, making it the most complete list so far for tracing the *A. oryzae* secretory machinery. Additional file 1 illustrates the workflow of detecting the *A. oryzae* secretory components, and detailed information can be found in Additional file 2: Table S1.

### Mapping the *A. oryzae* secretome to GO-Slim terms

**Figure 1:**
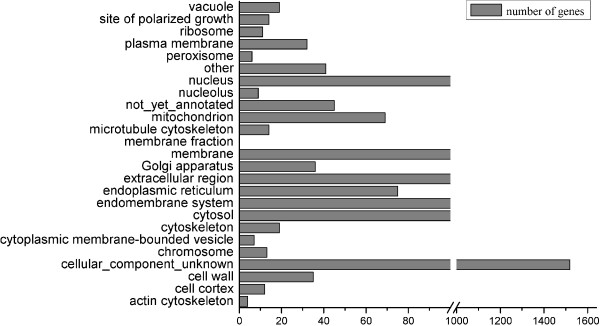
**Distribution of the putative *A. oryzae *secretome into GO-Slim terms.** The *A. oryzae* secretome was identified by FSD, and the GO-Slim classification was based on the facet of “Component” from AspGD.

Besides the α-amylase that was overexpressed, there are a large amount of native proteins performing diverse functions that need to pass through, and thereby compete for the resources in the secretory machinery. It would therefore be informative to monitor how these proteins respond to amylase overproduction. Most of these secretory proteins contain a N-terminal signal peptide as the key feature to enable their targeting and being processed via the secretory machinery [15,16]. In UniProt, only 118 out of 12,514 *A. oryzae* proteins were predicted to have signal peptides (experimentally or computationally). This number is unrealistically low as yeast (with ~6,000 ORFs) has been annotated to have secretome sizes varied from 560 (*S. cerevisiae* W303) to 918 (*S. cerevisiae* SC288C) genes [8]. Due to its larger genome size and more efficient secretion capacity, *A. oryzae* is expected to have many more proteins passing through the secretory pathway. Therefore we used the Fungal Secretome Database (FSD) [17] to define the *A. oryzae* secretome as it applies various algorithms (Method) to predict the clients on the secretory pathway. As a result, 2269 genes were identified to code for secretory clients according to FSD. These genes were further mapped to the *A. oryzae* GO Slim terms under the aspect “Component” using AspGD classification (Figure 1). The majority (58%) of the genes fall into the “cellular_component_unknown” group followed by “nucleus” (6.7%) and “membrane” (4.9%). Only 4.7% of genes are allocated to “extracellular region”, reflecting the fact that only a fraction of the proteins containing signal peptides are destined for the extracellular space [18]. Other proteins containing signal peptides may reside in the cytosol (4.3%), the ER (2.9%), the Golgi (1.4%), cell wall (1.3%), plasma membrane (1.2%) and elsewhere. The actual genes destined for the extracellular and plasma membrane may vary as many membrane proteins do not have N-terminal signal peptides as they use internal signals to integrate into the ER membrane [19] and some of the proteins without signal peptides potentially use unconventional secretory pathways [20].

### Construction of recombinant α-amylase over-producing strains of *A. oryzae*

In order to trace the secretory machinery response we constructed two novel *A. oryzae* α-amylase over-producing strains CF32 and A16. Together with an earlier reported high-level producer of α-amylase (CF1.1) [21,22], their performances were compared with the reference strain (A1560) [23], which produces basal level of amylase.

Multiple gene copies are frequently found to improve protein production [24], even though increasing copy numbers does not necessarily raise expression due to reasons such as saturation of transcription factors or pleiotropic effects of random integrations [3,25]. Regulation of protein expression mainly occurs on the transcriptional level. Here we relied on the endogenous transcription factors and applied two starch inducible promoters for expressing the TAKA amylase. The previous higher α-amylase producer CF1.1 contains additional copies of the TAKA amylase gene driven by TAKA promoter [23] compared to the reference strain A1560 [22]. To construct strains with even higher α-amylase production, one approach was to transform the reference strain A1560 with the TAKA amylase gene under the *A. niger* NA2 promoter [26], which has shown better effects on protein expression than the TAKA promoter [27]. Strain A16 was therefore constructed by transforming plasmid pLf1 harboring the TAKA amylase gene under the NA2 promoter into A1560 and selecting for higher producing clones. Another approach was to insert more copies of the TAKA amylase gene under the TAKA promoter into strain CF1.1 which already has elevated protein expression. Strain CF32 was constructed following this strategy by transforming plasmid pLf2, which has the α-amylase gene under the TAKA promoter, into strain CF1.1 (Additional file 3 shows how the strains were constructed, and Additional file 4 shows the primers, plasmids, strain, and transformation protocol).

### Strain characterization in batch cultivations

**Figure 2:**
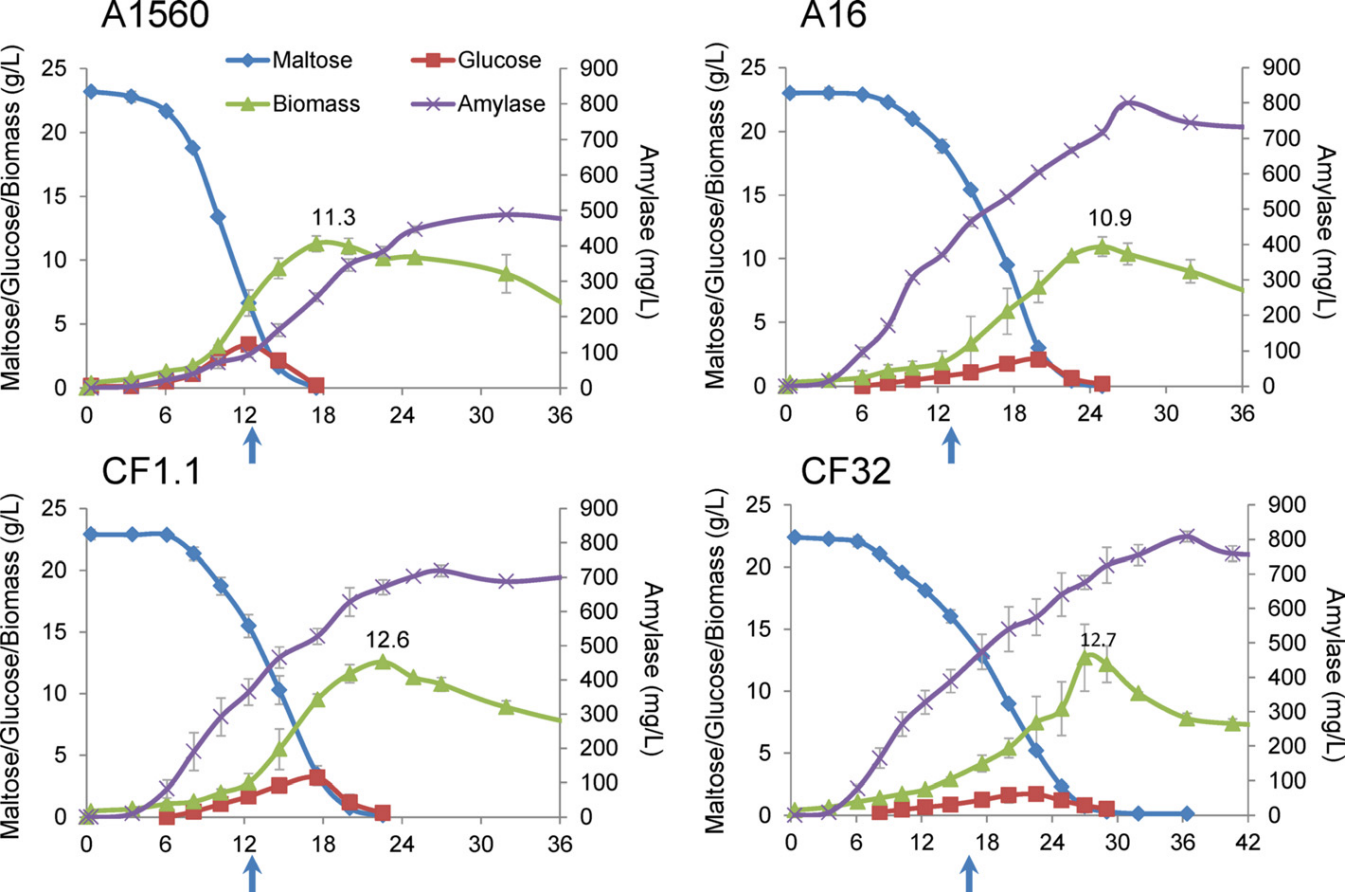
**Growth and enzyme production profiles of the α-amylase producing strains.** Left hand Y-axis represents the concentration of maltose (g/L), glucose (g/L) and biomass (g DCW/L), right hand Y-axis represents the titer of amylase (mg/L), and the X-axis represents fermentation time (hour). The arrows indicate the sampling time for microarray studies.

**Figure 3:**
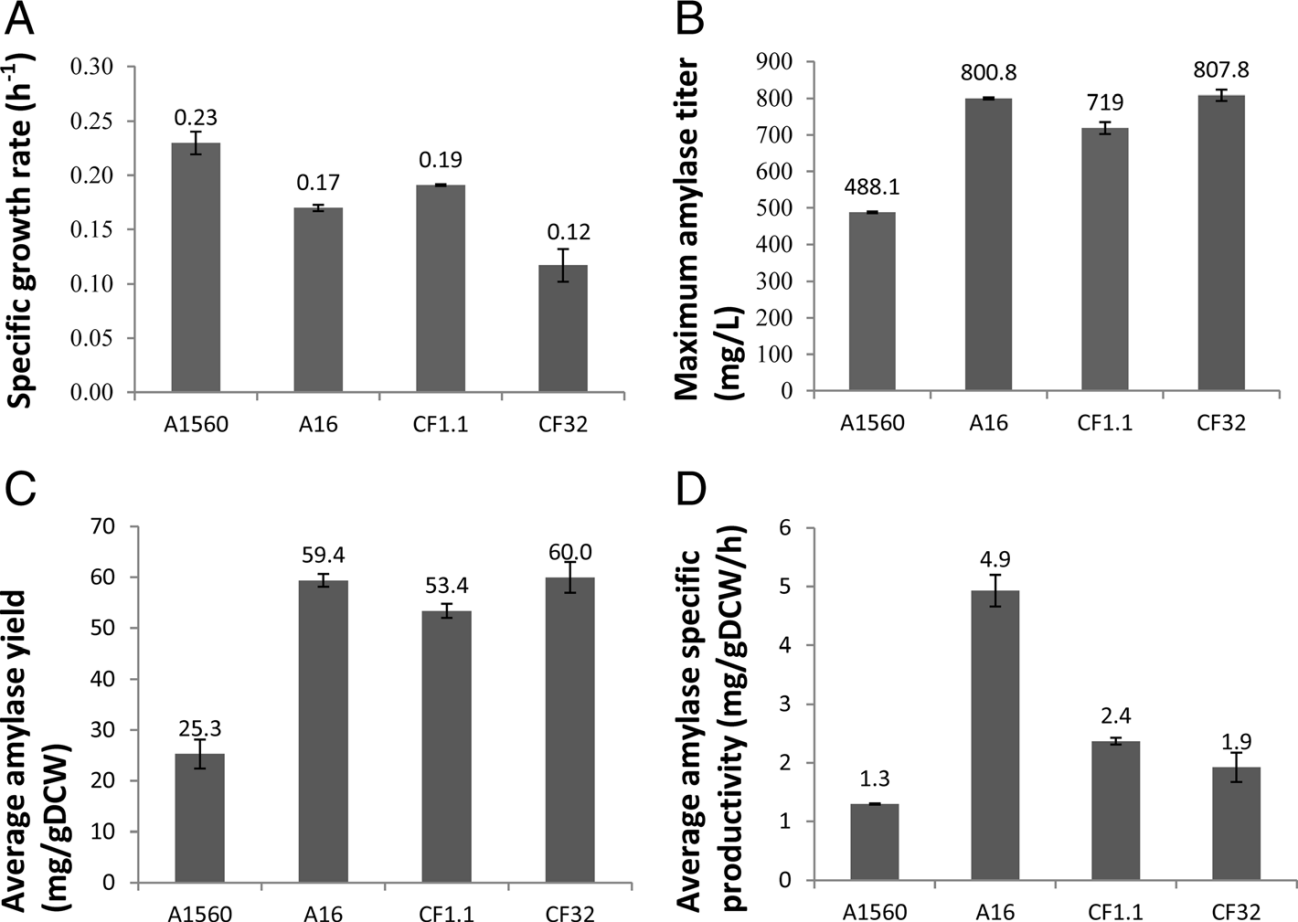
**Physiological parameters of the α-amylase producing strains in batch cultivations. (A)** Specific growth rate on maltose (h^−1^), **(B)** Maximum α-amylase titer (mg/L) **(C)** Average α-amylase yield (mg/g DCW), **(D)** Average specific α-amylase productivity (mg/g DCW/h). Error bars indicate the average of standard errors from independent duplicate fermentations.

The four strains were characterized in batch fermentations with maltose as carbon source as it is known to induce both promoters. Key kinetic variables were extracted from measurements of the sugar and biomass concentrations together with the enzymatic activity. The profiles of amylase production and cell growth (represented by maltose and glucose consumption and biomass generation) are shown in Figure 2. The specific growth rate (μ_max_), the average α-amylase yield on biomass (mg/g DCW), the average α-amylase productivity on biomass (mg/g DCW/h) and the final α-amylase titer (mg/L) are shown in Figure 3. It is observed that the wild type strain A1560 grew the fastest indicated by the highest μ_max_ of 0.23 h^−1^ (Figure 3A) and the fastest consumption of maltose and glucose before 18 hours (Figure 2). In contrast, strain CF32 grew the slowest, indicated by the μ_max_ of 0.12 h^−1^ and completed consumption of maltose and glucose after 30 hours. Strain CF1.1 and A16 had moderate specific growth rates of 0.19 h^−1^ and 0.17 h^−1^, respectively, corresponding to a maltose depletion at 22 and 24 hours, respectively. The average α-amylase yields at the time points where the maximum dry cell weights were reached were calculated by the α-amylase titers at that time point divided by the biomass produced. This data provides a measure of the relative production of secreted protein per unit of cell produced. If the number is high, it indicates that the cells are allocating resources towards α-amylase production and secretion rather than producing cellular proteins and other biomass components. The reference strain A1560 had the lowest yield (25.3 mg/g), followed by CF1.1 (53.4 mg/g), with A16 (59.4 mg/g) and CF32 (60 mg/g) having the highest yields. The maximum α-amylase titer in the fermentations showed similar trend as the average protein yield, being lowest for A1560 (488.1 mg/L), followed by CF1.1 (719 mg/L), and with the highest final titer for A16 (800.8 mg/L) and CF32 (807.8 mg/L) (Figure 2 and Figure 3B). In consideration of the time span for reaching the maximum average yield, strain A16 showed the highest specific productivity with 4.9 mg/gDCW/h followed by CF1.1 (2.4 mg/gDCW/h), CF32 (1.9 mg/gDCW/h) and finally the reference A1560 (1.3 mg/gDCW/h). This indicates that A16 stand out as the best production strain among the four as it has a high specific productivity, a high final titer and an acceptable specific growth rate.

### Global transcriptional response to α-amylase over-production

**Figure 4:**
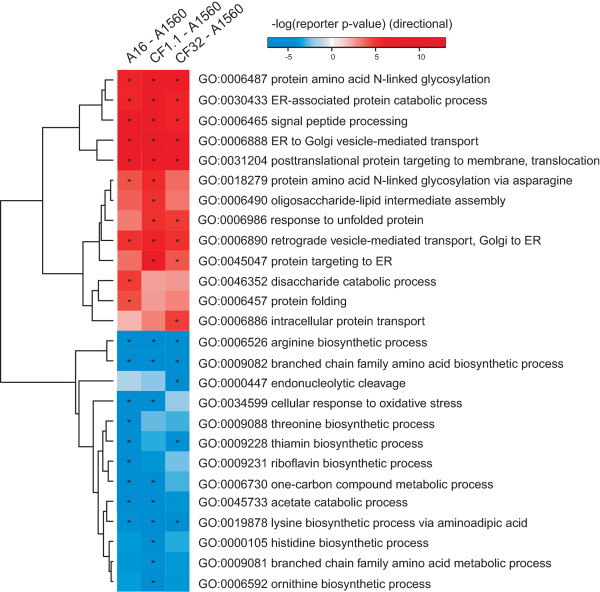
**Reporter GO-terms for the three α-amylase over-producing strains compared to the wild type A1560 strain.** Red color indicates that the genes belonging to the GO-term are up-regulated and blue color indicates down-regulation. The intensity corresponds to significance. GO-terms with reporter p-values smaller than 10^−4^ are indicated by asterisks.

In order to investigate the effect of α-amylase over production on the secretory pathway as well as on the whole cell metabolism we performed transcriptome analysis in the late exponential growth phase during batch fermentations. After normalization and statistical analysis we found 1212, 653 and 1709 genes to be differentially (adj. p-value < 0.05) expressed when comparing A16, CF1.1 and CF32 to A1560. Even though many differentially expressed genes have unknown functions due to poor annotations of the *A. oryzae* genome, we performed Reporter GO-term analysis [28,29] using the GO-term classification from AspGD where 7699 genes are classified into gene ontology terms. Shown in Figure 4, many genes with GO-term annotations related to protein secretion are significantly up-regulated in all three comparisons, including protein N-linked glycosylation, ER translocation and folding, signal peptide processing, ER to Golgi and the retrograde Golgi to ER vesicle trafficking *etc*. Other secretory related GO terms also appeared but in a strain dependent manner, e.g. response to unfolded protein only significant in CF1.1 and CF32 versus A1560. Several amino acid biosynthesis related GO terms are found down-regulated, in either three comparisons (arginine, branched chain family, lysine) or strain specific manner (histidine, ornithine) which might be due to the slower growth at the sampling time or the feedback inhibition on amino acid biosynthesis from overloaded ER stress. Down-regulation of amino acid biosynthesis has been observed in yeast in *HAC1* deletion strain overproducing heterologous proteins [30]. The unfolded protein response (UPR) in the recombinant *A. oryzae* strains may not be efficient enough to cope with the overloaded amylase and thus rendered similar consequence as in the *HAC1* deletion yeast strain that has impaired UPR. The transcriptional profiles of all the genes are described in Additional file 2: Table S2.

### Transcriptional response of the *A. oryzae* secretory machinery

In order to get a detailed mechanistic picture of the protein secretion response at the molecular level we mapped the gene transcriptional profiles to the reconstructed *A. oryzae* secretory machinery. With the most complete secretory component list, we were able to monitor the transcriptional response of the secretory machinery to the ultimate extend.

**Figure 5:**
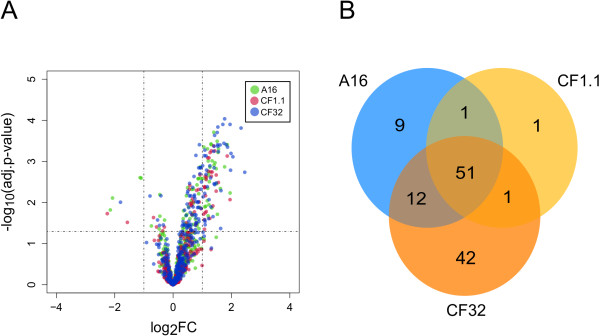
**The response of the secretory machinery in strain A16, CF1.1 and CF32 compared to A1560. (A)** Super imposed volcano plot showing the normalized expression for all component genes in all three recombinant strains compared with the wild type A1560. The genes are divided by dashed lines based on log fold change and significance and the most up-regulated genes (log FC ≥ 1 and –log10 (adj-pvalue) ≥1.3 (similar to adj-pvalue <0.05)) are shown on the top-left corner **(B)** The Venn diagram showing the distribution of the significantly changed secretory component genes in the three strains.

**Figure 6:**
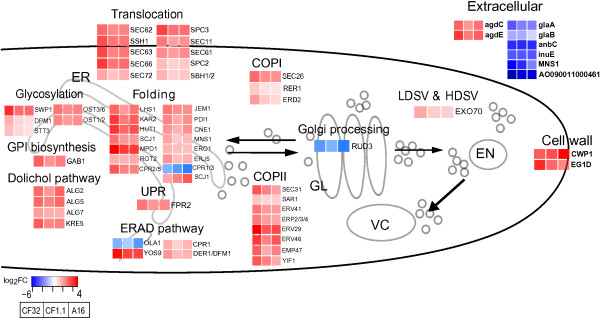
**Transcriptional profiles of the significantly changed genes in all three strains compared to the wild type.** Gene expressions are described based on log fold change compared to A1560, with red color indicating up-regulation and blue indicating down-regulation. Secretory component genes are written in normal font, whereas genes identified from the secretome analysis are in bold.

In line with the global transcriptional response (Figure 4), the volcano plot in Figure 5A shows that the machinery components in all three strains have similar transcriptional responses to α-amylase overproduction (Pearson correlation coefficient > 0.95). The transcriptional changes of all the machinery components are summarized in Additional file 2: Table S1. 51 out of the 369 components were found significantly changed (adj p <0.05) in all three comparisons (Figure 5B), among which 48 were up-regulated, and 3 were down-regulated. The transcriptional profiles of the significantly changed genes in all three strains are described in Figure 6 based on their classifications in the defined subsystems.

The TAKA amylase from *A. oryzae* contains one N-linked glycosylation site and four disulphide bonds [31]. Clearly, in comparison with the reference strain A1560, the subsystems responsible for the α-amylase PTMs, especially the components involved in ER processing (translocation, glycosylation, Dolichol pathway, Folding, UPR, ERAD, GPI biosynthesis, and trafficking between ER and Golgi) were significantly up-regulated, particularly in the CF32 strain which had the slowest growth compared to the other strains. Only two genes that are homologs of yeast *OLA1* and *CPR1/3* were down-regulated.

5 out of the 51 significantly changed *A. oryzae* secretory components were identified through blast search based on the yeast secretory machinery components [8] and have never been identified in *A. oryzae* from other studies. These novel *A. oryzae* secretory components are homologs to yeast *ERD2* (AO090102000650), *YOS9* (AO090023000334), *JEM1* (AO090020000010), *MNS1* (AO090003000057) and *GAB1* (AO090023000750) all of which showed significant up-regulation in response to α-amylase overproduction.

Erd2p mediates retrieval of the ER resident proteins from the Golgi through binding of the Erd2p receptor to the C-terminal peptide sequence HEDL on soluble ER resident proteins such as proteins encoded by *KAR2*, *PDI1*, *ERO1*, *FKB2* and many more in yeast [32]. ER retrieval meditated by Erd2p has been suggested as an non-essential process as Ire1p was reported to share functional redundancy to maintain normal levels of ER residential proteins in yeast [33]. The transcriptional levels of the *A. oryzae IRE1* homolog were not changed in all three amylase overproducing strains compared to the wild type which may indicate that *A. oryzae* has a different mechanism to retrieve ER residential proteins than yeast, and the *A. oryzae ERD2* homolog seems to play a more important role than the *IRE1* homolog.

Ire1p is also well known to splice the *HAC1* mRNA which once transcribed will trigger the UPR to alleviate folding stress in the ER [34]. Genes encoding folding chaperones such as *A. oryzae* homologs of *CNE1*, *KAR2*, *PDI1*, *MPD1*, *FKP2* and the *KAR2*’s co-chaperones *SCJ1* and *JEM1* were all significantly up-regulated, reflecting that the UPR was actively turned on in all strains compared with the wild type. However, both the mRNA levels of the *HAC1* homolog and the *IRE1* homolog were not differentially changed indicating that the *IRE1*-mediated *HAC1* splicing is not the only mechanism for activating UPR in *A. oryzae*. Actually, an *IRE1*-independent surveillance mechanism that monitors protein folding in the ER has been indicated in yeast [35] and in metazoan cells. There are already three mechanistically distinct pathways, mediated by *IRE1*, *ATF6* and *PERK* respectively, known to operate in parallel to activate UPR in mammalians [36]. A study overexpressing membrane protein in *A. niger* showed the mRNA level of BipA (encoded by *KAR2*) was elevated while no truncated *hacA* transcript was detected [37]. With transcriptomic data on genes encoding *A. oryzae* homologs of *HAC1*, *IRE1* and the UPR activated ER chaperones, our analysis further implied that the *IRE1*-mediated *HAC1* splicing is not the sole mechanism for activating UPR in *A. oryzae* and it may share the *IRE1*-independent mechanism with yeast, metazoan or mammalian.

Another interesting observation is that some of the yeast components were mapped to more than one *A. oryzae* ORFs whose transcriptional level can differ significantly. For example, three *A. oryzae* homologs were found for yeast *CPR1*, with AO090023000811 significantly up-regulated in three comparisons, AO090120000486 significantly down-regulated, and AO090120000215 not changed; *DER1* homolog AO090701000076 significantly up-regulated in three comparisons while no change was found for AO090701000551; *ERD2* homolog AO090102000650 significantly up-regulated while no change was found for AO090026000646. Homologs of the same yeast gene can also behave similarly, as for those of the folding chaperone *ERJ5*, AO090003000036 and AO090011000874 were both significantly up-regulated in the three comparisons. These phenomena reflect that the secretory machinery components are duplicated in *A. oryzae*. The increased number of components might be associated with more sophisticated functions or regulations in the *A. oryzae* secretory pathway than in yeast.

We did not see significant transcriptional changes on post ER processes, which suggest that they might be more regulated on protein level or they are regulated on transcription level but to a much lesser extent.

### Transcriptional response of the secretome to amylase overproduction

**Figure 7:**
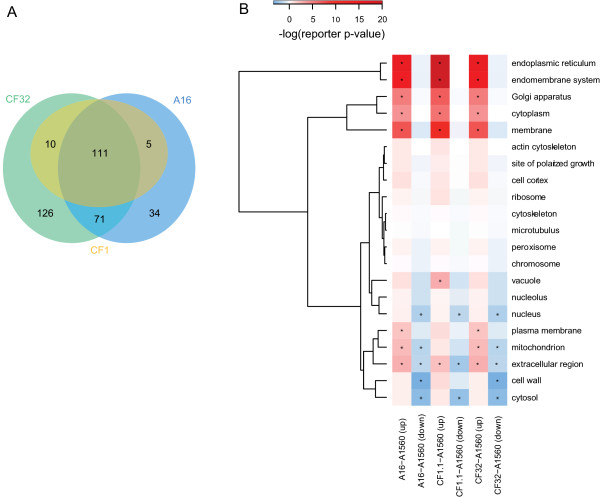
**Transcriptional profiles of the significantly changed secretomes. (A)** Venn diagram showing distribution of the significantly changed secretome genes. **(B)** Reporter GO-Slim terms for the secretome genes that have annotated localizations. Red color indicates up-regulation of the genes in the particular GO-Slim terms and green color indicates down-regulation of the genes in the corresponding GO-Slim terms. An asterisk indicates the reporter p-value for the GO-term is lower than 0.05.

The transcriptional response of the secretome to a specific recombinant protein is important in a sense that if the target protein overloads the secretory machinery, the cell probably needs to change its secretome profile to adapt to the processing capacity of the secretory machinery. To examine this idea we compared the expression profiles of our defined secretome of *A. oryzae* in response to amylase overproduction in the three recombinant strains compared to the wild type A1560 (Additional file 2: Table S3). 357 out of the 2269 putative genes in the predicted secretome were significantly changed, with 111 genes found in all three comparisons, 126 genes found uniquely in CF32 and 34 genes found uniquely in A16 (Figure 7A). We performed Reporter GO-term analysis [28,29] using the GO-Slim classification based on component (Figure 7B) for the 496 secretome genes that have a localization annotation in AspGD (Additional file 2, Table S4).

According to the GO-Slim enrichment analysis, genes encoding secretory proteins localized to the ER, the Golgi apparatus, the cytoplasm and the membranes were distinctively up-regulated in all three comparisons, most of which have been identified as *A. oryzae* secretory components (Additional file 2: Table S1). Several additional genes such as AO090026000662 (*SSP120*), AO090023000124 (*VPS66*) and AO090003001323 (*PHO88*) were also detected in these localizations showing significant up-regulation.

Genes encoding proteins secreted to the extracellular region, the vacuole, the cell wall were either significantly up- or down- regulated which may be informative for further investigation. Interestingly, two genes predicted to reside in the fungal cell wall namely AO090701000717 (homolog of *A. nidulans eglD*) and AO090011000119 (homolog of *A. niger cwpA*) were significantly up-regulated in all three strains. EglD is a putative endoglucanase discovered in the conidial cell wall of *A. nidulans* carrying an expansin like domain [38]. Expansins exhibit wall loosening activity and are involved in plant cell expansion and other developmental events. The expansins are highly conserved among plants and fungi [39]. EglD in *A. nidulans* has been indicated to be involved in fungal cell wall remodeling during germination [38]. The significant up-regulation of its *A. oryzae* homolog AO090701000717 in the amylase overproduction strains might be the consequence of amylase overloading to the secretory pathway. Remodeling of the cell wall through *eglD* up-regulation may possibly help to loose cell wall structure and facilitate amylase secretion. There is no direct explanation for the up-regulation of the mannoprotein cwpA, however, since the surface properties of fungi are primarily determined by the presence of cell wall mannoproteins [40], the expression of *cwpA* may also be altered in response to *eglD* up-regulation to facilitate the remodeling of the fungal cell wall and ultimately the protein secretion.

Transcriptional down-regulation on *glaA* encoded glucoamylase (EC 3.2.1.3) has been reported *in A. niger* in response to ER stress induced by DTT [41]. Two *A. niger* and *A. nidulans glaA*/*glaB* homologs AO090003000321 and AO090010000746 were also found down-regulated in strain A16 and CF32 that supposed to have higher ER stress due to higher amylase yields and slower growths than CF1.1. Both TAKA-amylase and glucoamylase expressions are regulated by AmyR which activates their transcriptions in the presence of starch or maltose [42,43]. The glucoamylases may therefore serve as competitors to TAKA-amylase for not only transcription factors but also for secretory machinery. Other genes that have an extracellular region localization such as AO090011000461 (uncharacterized), AO090138000055 (homolog to *A. niger anbC*, endo-1,5-alpha-L-arabinosidase), AO090701000400 (homolog to *A. niger inuE*, sucrose alpha-glucosidase) and AO090003000476 (*A. niger* and *A. nidulans mns1*, 1,2-alpha-mannosidase) were all down-regulated in three comparisons. As also extracellular enzymes these gene products may very likely go through the same secretory pathway as the TAKA-amylase [44]. To efficiently utilize nutrient and cellular resources, the cells may slow down the expression of the competitor genes and divergent resources to synthesize and secrete the TAKA-amylase enforced for overexpression. As exemptions, genes AO090026000034 (homolog to *A. niger agdC* and *A. nidulans adgE*) and AO090102000559 (homolog to *A. niger agdE*) encoding alpha-glucosidase (EC 3.2.1.20) were significantly up-regulated especially in A16 and CF32. Since this enzyme directly hydrolyzes maltose to glucose, the up-regulation of the genes should be the cellular response to digest the carbon source maltose.

Isoenzymes performing analogous functions might be regulated differently according to their localizations. For example, more than one 1, 2-alpha-mannosidases (EC 3.2.1.24) were found in *A. oryzae*. AO090003000476 encoding protein has a predicted extracellular localization and thus its transcription was down-regulated to leave resources for TAKA-amylase. In contrast, AO090003000057 encoded protein is important for performing N-glycosylation and folding in the ER and thus the gene was significantly up-regulated. AO090003001225 did not have a transcriptional change and its localization is unclear.

### Co-expression analysis of genes changed uniquely in CF32-A1560 and A16-A1560 comparisons

**Figure 8:**
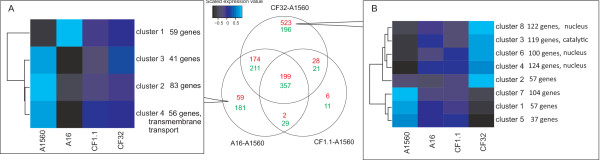
**Co-expression analysis of the genes changed uniquely in A16 and CF32.** The venn diagram in the middle shows the number of the transcriptionally up-regulated (red) and down-regulated (green) genes (FDR < 0.05) in the three α-amylase overproducing strains compared to the wild type. **(A)** The clustergram showing the co-expression clustering of the genes changed significantly in A16 compared to A1560 but not in the other comparisons. **(B)** The clustergram showing the clusters of genes significantly changed in CF32 compared to A1560, but not in other comparisons.

A16 and CF32 grew relatively slower than CF1.1, while they had higher amylase yields and final titers (Figure 3). In order to look into the strain-specific responses of these two strains, we performed clustering analysis for i) the genes that were significantly changed in expression in the A16 vs. A1560 but not in any other comparisons and ii) the genes that change significantly when comparing CF32 vs. A1560 but not in any other comparisons (Figure 8). The genes changed uniquely in the comparison of A16 vs. A1560 was grouped into 4 clusters, where the genes in cluster 1 were up-regulated and the genes in cluster 2, 3 and 4 were down-regulated compared to A1560 (Figure 8A). From the down-regulated gene clusters we identified three sequence motifs that are enriched in the upstream regions of the genes in the clusters. 135 sites contain AAGAA, 27 sites contain CCCCT, and 29 sites contain ACTACTA, which are consensus binding sites for yeast transcription factors Azf1p, Msn2p/Msn4p, and Smp1p respectively. Msn2p/Msn4p and Smp1p are known stress response regulators in *S. cerevisiae*[45]. The down-regulation of the genes regulated by them might indicate that the A16 strain has less cellular stress compared with the other strains, which could be one of the reasons for its better performance. The genes changed uniquely in the comparison of CF32 vs. A1560 were grouped into 8 clusters where clusters 2, 3, 4, 6 and 8 contain up-regulated genes and 1, 5, 7 contain down-regulated genes (Figure 8B). GO-term over-representation using a hypergeometric test for the genes in the up-regulated clusters showed that these genes have DNA-binding and catalytic functions, which could be explained by the higher amylase copy numbers in the CF32 strain as it may request for higher transcriptional efficiency. Motif AAAAGAAAA, binding site of yeast Azf1p, was also identified in the down-regulated clusters. Based on the Azf1p functions in *S. cerevisae*, similar roles as to activate transcription of genes involved in growth and carbon metabolism or in maintenance of cell wall integrity could be expected. The down-regulation of these genes indicated certain changes happened in these facets in response to amylase overproduction. The genes belonging to different clusters and the identified putative sequence motifs on the upstream regions are listed in Additional file 2: Table S5.

## Conclusion

In this work, by providing a far more complete secretory component list of *A. oryzae* we were able to monitor the whole secretory pathway in response to α-amylase overproductions at the molecular level. The roles of many predicted secretory machinery components were verified with their transcriptional changes. The defined *A. oryzae* component list offers a better platform to trace the secretory machinery responses on genome (gene variation), transcriptome, and proteome levels. From this analysis we could discuss several interesting mechanisms based on the transcriptomic data of the identified components, such as the *ERD2* mediated retrieval of the ER residential proteins may be more dominant in *A. oryzae* than in yeast and there could be an *IRE1*-independent system to trigger the UPR response in *A. oryzae.*

Additionally, this study generated a list of targets for genetic manipulation. For example, overexpressing the up-regulated *eglD* and *cwpA* encoded cell wall proteins, and knocking out the genes encoding extracellular proteins competing for the secretory pathway may help to increase protein secretion in this industrially important fungus.

## Method

### Detecting the secretory components and subsystems

For detecting the components we used the one-to-one ortholog mapping with identity >80%. For more divergent components we used PSI-blast [9] to collect the best hits with E-Value < 0.05.

### Defining the secretome

The Fungal Secretome Database (FSD) [17] integrated nine prediction programs based on their abilities to predict i) whether a protein contains a signal peptide, ii) contains transmembrane helix, iii) has a nucleus localization signal, iv) secretes via non-classical pathway and v) where the protein probably resides. Using this database and we predicted 2269 putative genes in *A. oryzae* genome to be potentially secreted.

### Transcriptome response of the secretory machinery

The volcano plot was generated in R (http://www.r-project.org/) to assess the transcriptional changes of the genes involved in the *A. oryzae* secretory machinery in all three strains compared to A1560. The significantly regulated genes in all three strains were included to make the venn diagram.

### Batch fermentation and sampling

Pre-cultures were prepared by inoculating 10^9^ spores harvested in 5 mL of Tween 80, 0.1% from mycelium that had been grown for 6 days on Cove-N-Gly plate at 30°C for 6 days into 100 mL of G2-Gly medium and shaking at 250 rpm at 30°C for 24 hours. About 40–45 mL of pre-culture was inoculated into 1.2 L of BCM medium in 2.7 L bioreactors (Z61101C006, Applikon, Netherlands). The temperature was maintained at 34°C and the air aeration at 1.2 vvm during the whole fermentation. The stirring speed was kept at 800 rpm for the first 4 hours and later increased to 1,000 rpm. The pH was controlled at 6 by 10% (v/v) of H_3_PO_4_ or NH_3_ · H_2_O. All fermentations were performed in biological duplicates.

5 mL of culture broth was filtered through a 0.45 μm filter membrane and then dried at 95°C for 24 hours and cooled down in a desiccator. The dry cell weight was calculated by measuring the increased weight of the dried filter. Clear supernatant was obtained by centrifugation and filtration as mentioned above and then loaded to a HPX-87G column (BIORAD, USA) on a Dionex Ultimate 3000 HPLC (Dionex Softron GmbH, Germany) to measure the concentrations of extracellular metabolites, including glucose, ethanol, glycerol pyruvate, *etc*. Samples were run with a flow rate of 0.6 mL/min at 65°C using 5 mM H_2_SO_4_ as mobile phase.

### Microarray data analysis

#### RNA extraction

Total RNA was extracted using the RNeasy Mini Kit (QIAGEN) according to the protocol for purification of total RNA from filamentous fungi. The quality and quantity of the total RNA was determined by an Agilent 2100 bioanalyzer using RNA 6000 Nano Kit (Agilent Technologies). Total purified RNA was stored at −80°C until further microarray processing. The total RNA was converted to biotin labeled cDNA as described previously and hybridized onto the *Aspergillus* GeneChip (3AspergDTU) [46]. The arrays were scanned to obtain raw CEL-files.

#### Microarray preparation and processing

The CEL-files were preprocessed using Bioconductor [47] and R version 2.12.0. The Affymetrix chip description file (CDF-file) was obtained from the microarray developers and imported to R using the Bioconductor package makecdfenv. The raw data were normalized using Probe Logarithmic Intensity Error (PLIER) normalization [48] using only perfect match probes (pm-only). The moderated t-statistic was applied to identify pairwise differences in gene expression between each of the three amylase producing strains and the A1560 strain. The p-values were corrected for multiple testing using Benjamini-Hochberg’s method [49]. Genes with a corrected p-value (adj.P) lower than 0.05 were considered to be differentially expressed between two conditions.

#### Reporter GO-term analysis

The gene ontology classification (GO-terms) for the *A. oryzae* genes was downloaded from the AspGD [7] in October 2012. The gene IDs for the genes on the microarray were checked for consistency with the gene annotation presented in AspGD and we found that some of the probes on the microarray were named using the gene alias (alternative gene IDs) in AspGD. The corrected gene annotation list has 7699 genes associated with one or more gene ontology-term. The reporter features algorithm [28] was used to score each GO-term based on the significance of the genes belonging to this ontology term. To identify GO-terms with mainly up- or down-regulated genes the reporter algorithm was used twice, once with only up-regulated genes as input and once with only down-regulated genes as input. The analysis was performed using functions in the PIANO R-package [29]. We also performed the same reporter GO analysis, but only using genes defined in the list of the *A. oryzae* secretome described in this paper and using the GO-Slim annotation for cellular compartment (Components) downloaded from AspGD.

#### Integrated analysis

We used our transcriptome data to extract the normalized average expression data (log_2_FC) for the secretory components which were differentially expressed in all three strains (A16, CF1.1 and CF32) compared to the wild type (A1560) with adjusted p-value < 0.05. The PIANO package was used to produce the heatmap of the machinery components [29].

#### Co-expression analysis

The genes that changed uniquely in A16 vs A1560 and CF32 vs. A1560 comparisons were clustered based on correlation in the following way. Firstly the expression values were transformed to range between −1 and 1 based on the correlation matrix between all genes. Secondly the scaled expression values were clustered using affinity propagation [50] and finally the centroids of the clusters were clustered again using hierarchical clustering.

### Ethics

We claim that there is no ethics related issue in this work.

## Abbreviations

PTMs: Post translational modifications; UPR: Unfolded protein response; ERAD: ER associated degradation; OST: Oligosaccharyltransferase; GPI: Glycosylphosphatidylinositol; FSD: Fungal secretome database; AspGD: *Aspergillus* genome database; GO: Gene ontology.

## Competing interests

The authors declare that they have no competing interests.

## Authors’ contributions

LL constructed the strains, performed all the experimental work and analyzed the data. AF enriched the protein secretory component list. TÖ performed data analysis. CH supervised the strain construction. JN conceived the project and supervised the work. LL, AF, TÖ and JN wrote the paper. All authors contributed with editing of the paper. All authors read and approved the final manuscript.

## Supplementary Material

Additional file 1 **Workflow for detecting the *A. oryzae *** **secretory components.** The *A. oryzae* secretory components are identified from four sources. 1st-1: homologs (Inparanoid+besthits) of yeast secretory components identified in Feizi, *et al.*[8]; 1st-2: homologs (Iterative PSI-blast) of yeast secretory components identified in Feizi, *et al.*[8]; 2nd: *A. oryzae* secretory components from Wang *et al.*[10]; 3rd: homologs (Inparanoid+besthits) of *A. niger* secretory components identified by Oliveira *et al.*[11]; 4th: *A. oryzae* SNARE protein identified by Kuratsu *et al.*[12]. The numbers in brackets specify the number of components identified from each sources. Components included in the final list are highlighted in red. Overlapping components are in black. Click here for file

Additional file 2: Table S1 *A. oryzae* secretory components and their transcriptional responses to amylase overproduction. **Table S2.** Global transcriptional changes in response to amylase overexpression in strains A16, CF1.1 and CF32 compared to A1560. **Table S3.***A. oryzae* secretome extracted from FSD database and their transcriptional responses to amylase overproduction. **Table S4.***A.oryzae* secretome genes that have a predicted localization based on GO-Slim analysis. Table S5. Gene clusters changed uniquely in A16 vs A1650 or in CF32 vs A1560 + enriched TF binding motifs. Click here for file

Additional file 3 **Construction of *A. oryzae *** **α-amylase overproducing strains.** Starting strain A1560 was co-transformed with a TAKA amylase driven by the TAKA promoter and a selection vector bearing amdS selection marker to make strain CF1.1. Strain A16 was constructed by transforming a vector containing the TAKA amylase under the NA2 promoter together with an amdS selection vector. Strain CF32 was made by transforming a vector harboring TAKA amylase genes under the TAKA promoter and the *Bar* gene for selection into strain CF1.1. Click here for file

Additional file 4 **Strains, Plasmids and Media; α-amylase quantification; Transformation protocol for *Aspergillus *** **using top agar; Primers used in this study.** Click here for file
