# Supplementary figures and images for: Genome-scale analysis of the high-efficient protein secretion system of Aspergillus oryzae
Source: BMC Syst Biol. 2014 Jun 24;8:73. doi: 10.1186/1752-0509-8-73 (PMC4086290; doi:10.1186/1752-0509-8-73)

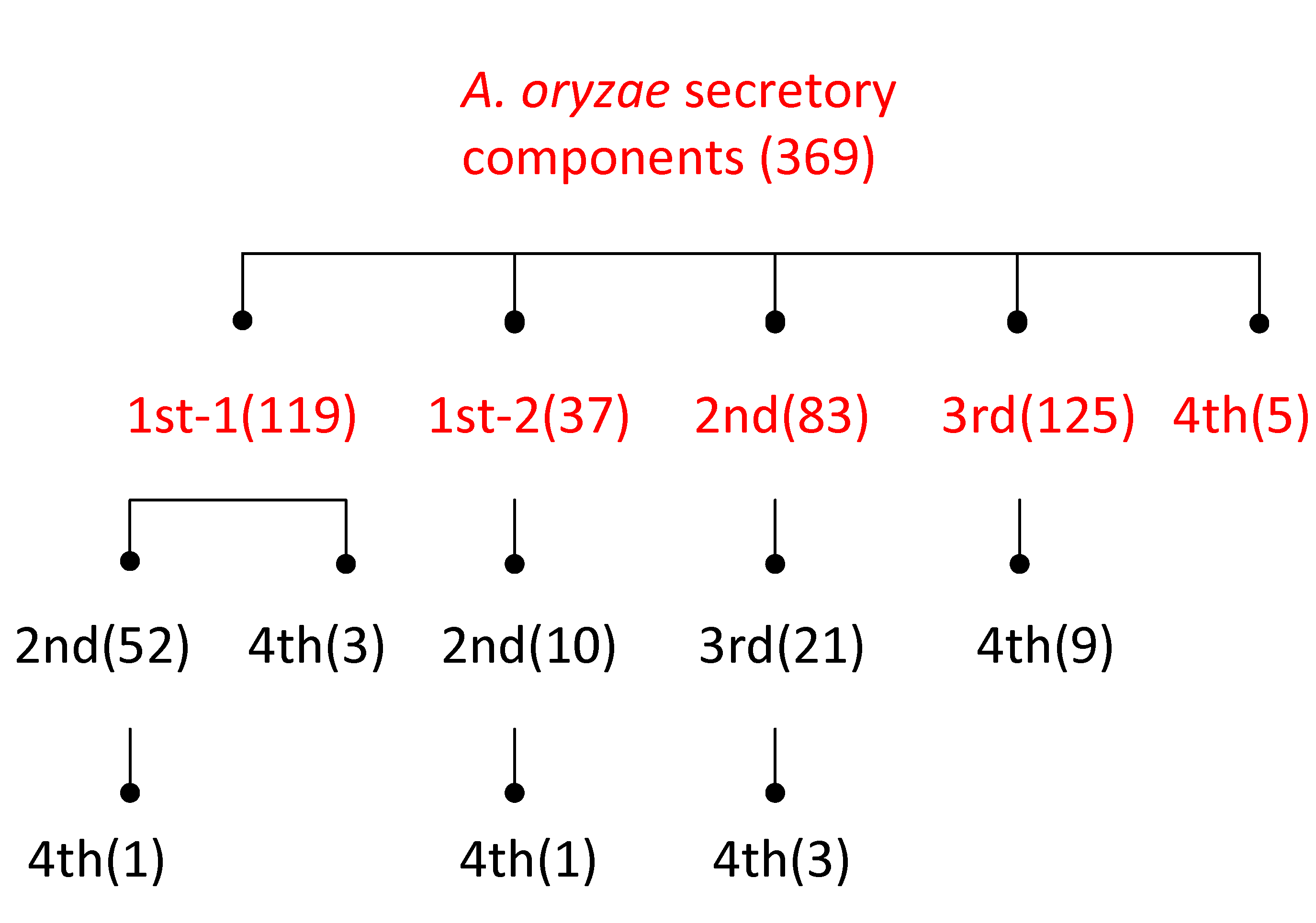

Supplement: Additional file 1 — Workflow for detecting the A. oryzae secretory components. The A. oryzae secretory components are identified from four sources. 1st-1: homologs (Inparanoid+besthits) of yeast secretory components identified in Feizi, et al.[8]; 1st-2: homologs (Iterative PSI-blast) of yeast secretory components identified in Feizi, et al.[8]; 2nd: A. oryzae secretory components from Wang et al.[10]; 3rd: homologs (Inparanoid+besthits) of A. niger secretory components identified by Oliveira et al.[11]; 4th: A. oryzae SNARE protein identified by Kuratsu et al.[12]. The numbers in brackets specify the number of components identified from each sources. Components included in the final list are highlighted in red. Overlapping components are in black. [file 1752-0509-8-73-S1.tiff]

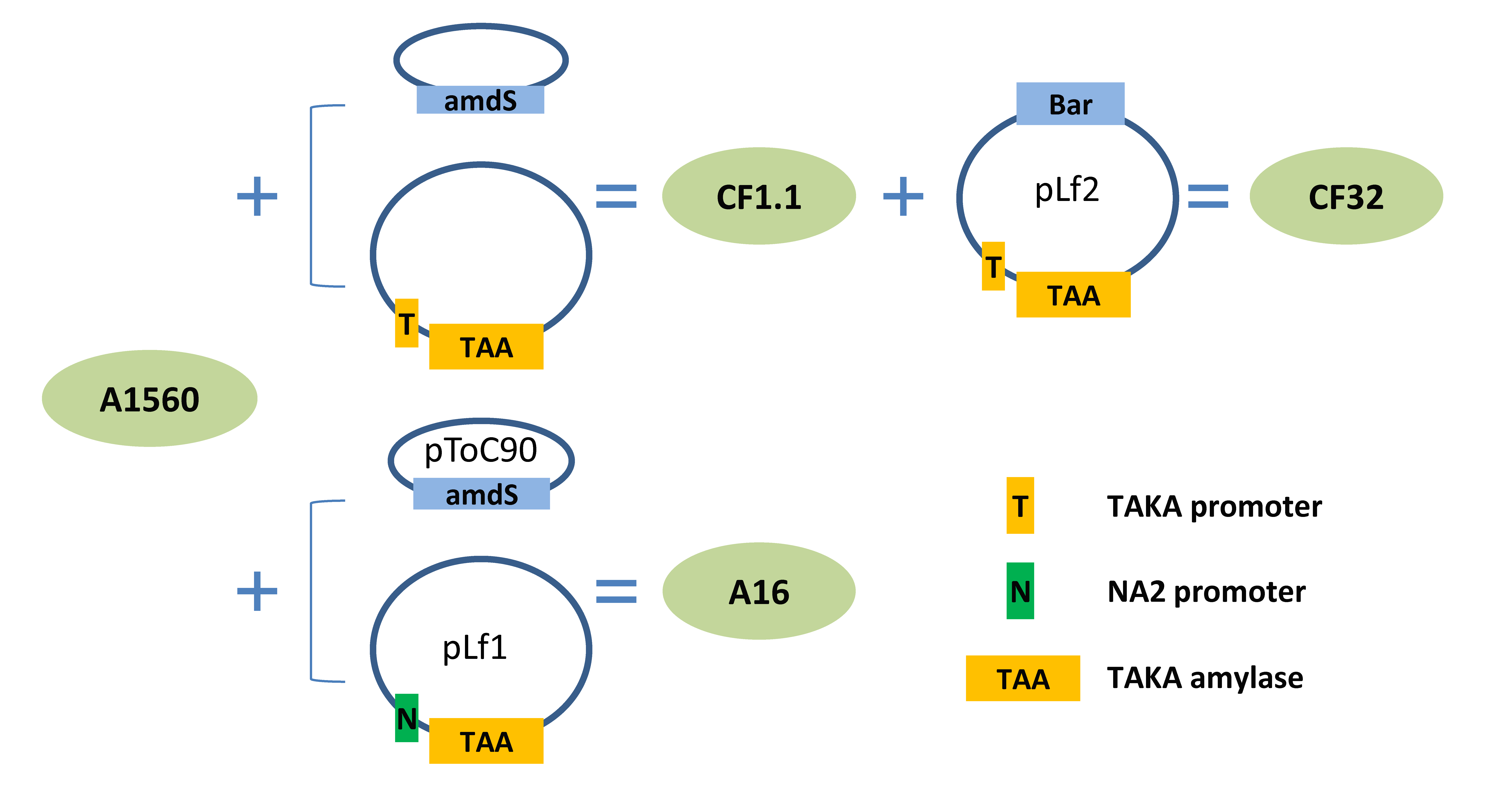

Supplement: Additional file 3 — Construction of A. oryzae α-amylase overproducing strains. Starting strain A1560 was co-transformed with a TAKA amylase driven by the TAKA promoter and a selection vector bearing amdS selection marker to make strain CF1.1. Strain A16 was constructed by transforming a vector containing the TAKA amylase under the NA2 promoter together with an amdS selection vector. Strain CF32 was made by transforming a vector harboring TAKA amylase genes under the TAKA promoter and the Bar gene for selection into strain CF1.1. [file 1752-0509-8-73-S3.tiff]
