# Supplementary material for: Genome-scale analysis of the high-efficient protein secretion system of Aspergillus oryzae
Source: BMC Syst Biol. 2014 Jun 24;8:73. doi: 10.1186/1752-0509-8-73 (PMC4086290; doi:10.1186/1752-0509-8-73)
Supplement: Additional file 4 — Strains, Plasmids and Media; α-amylase quantification; Transformation protocol for Aspergillus using top agar; Primers used in this study. [file 1752-0509-8-73-S4.doc]

**Strains, Plasmids and Media**

The starting *A. oryzae* strains A1560 and CF1.1 and plasmids pToC52, pCaHj447, pJaL1097 and pToC90 were kindly provided by Novozymes A/S. Plasmid constructions were performed using *Escherichia coli* DH5α (Bethesda Research Laboratories) following standard molecular biology techniques. *A. oryzae* transformations were performed following the top agar protocol described in Additional file 6. All primers are recorded in Additional file 7.

Plasmid pLf1 was built by inserting the TAKA amylase fragment amplified by primers TAKA_amy_fw/ TAKA_amy_re from plasmid pToC52 with added Kazak sequence at the 5’ end and *BamH*I and *Xho*I sequences at the 5’ and 3’ ends respectively into pCaHj447 through Infusion cloning (In-Fusion HD Cloning Kit, Clontech). Plasmid pLf1 was co-transformed with pToC90 (harboring *amdS* gene from *A.nidulans*) into A1560 and selected on amdS selective plate. Plasmid pLf2 was constructed by inserting *bar* gene fragment amplified by Bar_fw/Bar_re into the *Sph*I-*BssH*II cassette of the TAKA amylase expression vector pToC52. Plasmid pLf2 was transformed into CF1.1 with BASTA selection. The transformants strains were screened by α-amylase expression checked on SDS-PAGE. A16 performed the best among the A1560 transformants, and CF32 ranked the highest among the CF1.1 transformants. The TAKA amylase gene is placed after TAKA-amylase promoter in plasmid pLf1 and after *A. niger* NA2 promoter in plasmid pLf2.

Spore propagation agar plate (Cove-N-Gly) and pre-culture medium (G2-Gly) were made according to Vongsangnak W *et al* . The batch cultivation medium (BCM) comprises of 22.5 g/L maltose, 1 g/L MgSO_4_·7H_2_O, 1.5 g/L KH_2_PO_4_, 7.3 g/L (NH_4_)_2_SO_4_, 1 g/L NaCl, 0.1 g/L CaCl_2_·H_2_O, 0.5 mL/L Pluronic PE-6100 and 0.5 mL/L Tracer (14.3 g/L ZnSO_4_·7H_2_O, 2.5 g/L CuSO_4_·5H_2_O, 0.5 g/L NiCl_2·_6H_2_O, 13.8 g/L FeSO_4_·7H_2_O).

## Assay for α-amylase quantification

Culture supernatants were collected by centrifuging 1 ml of cultural broth at 10,000 g for 5 min and filtrating through 0.45 µm filters. 800 µl of clear supernatant was mixed with 100 µl of 5.5 mM of NaN3 in 0.1 M of HCl and placed in 4 ^o^C before measurement. α-amylase activity was measured using the Ceralpha kit (Megazyme, Ireland) with α-amylase originated from *A. oryzae* (Sigma 82650, USA) as standard. Since the standard α-amylase is a fungal extract which is not pure protein, a calculation addressed in Liu Z *et al*  was used to convert the α-amylase activity (U) to its corresponding mass (g).

# Transformation protocol for Aspergillus using top agar

An agar slant (COVE-N-Gly medium) was inoculated with spores of the strain in question, and the strains were grown at 37°C for approx 6 days.

* 10 - 20 ml of Sucrose medium was added to the slant, and the spores were suspended by vortexing. The spore suspension was transferred to a polycarbonate shakeflask (500 ml) containing 100 ml sucrose medium and 10mM NaNO3.The flask was incubated at 30°C for 24 hr (200 rpm).

* The mycelium was collected by filtration through mira cloth, and was washed using 200 ml 0.6 M MgSO_4_. The remaining liquid was squeezed out of the mycelium e.g. using a plastic pipette.

* 1-2 g of the mycelium was transferred to a small (100 ml) polycarbonate flask containing:
 75-150 mg Glucanex (Novozymes)
 10 ml 1.2 M MgSO_4_
 100 ul 1 M NaH_2_PO_4_ pH 5.8
 and the mycelium was suspended.
 1 ml of 12 mg/ml BSA (sterile filtered) was added.

* The suspension was incubated at 37°C for 1/2-2 hr, and the protoplasting was monitored frequently by microscopy.

* The protoplast suspension was filtered through mira cloth into a 25 ml NUNC tube and the suspension was overlaid with 5 ml ST and the protoplasts were banded by centrifugation (2500 rpm / 1350 g, 15 min, slow acc.). The interface band of protoplasts was recovered using pipette and transferred to a fresh tube.

* The protoplasts were diluted with 2 volumes of STC followed by centrifugation (2500 rpm / 1350 g, 5 min). The protoplasts were washed twice with 5 ml STC (using resuspension and centrifugation), and then resuspended in STC to a concentration of approx 5 x 10^7^ protoplasts/ml.

* For each transformation, the transforming DNA was added at the bottom of a 14 ml Falcon tube, and 100 µl of protoplasts were added. 300 µl of PEG was added, and the tube was gently mixed by hand. After 20 minutes of incubation (RT), 6 ml top agar at temperature of 50ºc was added and immediately the suspension was poured on to the selective plate.

* The plates were incubated at 37°C until transformants were clearly visible and started to sporulate.

**Media and solutions**

Trace element solution: 40 mg Na_2_B_4_O_7_10H_2_O

400 mg CuSO_4_5H_2_O

800 mg FeSO_4_7H_2_O

800 mg MnSO_4_2H_2_O

800 mg Na_2_MoO_4_2H_2_O

8 g ZnSO_4_7H_2_O

H_2_O to 1L

Salt solution: 26 g KCl

26 g MgSO_4_7H_2_O

76 g KH_2_PO_4_

50 ml trace element solution

H_2_O to 1L

Sucrose medium: 20 ml salt solution.

342 g sucrose.

H_2_O to 1L

Autoclave and then add:

10 mM NaNO3

ST 0.6 M sorbitol

100 mM Tris/HCl pH 7.0

STC 1.2 M sorbitol

10 mM CaCl_2_

10 mM Tris/HCl pH 7.5.

PEG 60% (W/V) PEG 4000 (BDH) (60-65°C)

10 mM CaCl_2_

10 mM Tris/HCl pH 7.5

amdS selective plates 20 g agar

20 ml Salt solution

342 g sucrose

H_2_O to 1L

Autoclave

10 ml 1M Acetamide

15 ml 1M CsCl

Top agar 500 ml Sucrose medium

5 g Low melting agarose

10 ml 20 mM Tris pH 7.5

Autoclave

Basta selective plate

Christensen T, Woeldike H, Boel E, Mortensen SB, Hjortshoej K, Thim L, Hansen MT (1988) High level expression of recombinant genes in *Aspergillus oryzae*. *Nature Biotechnology* **6:** 1419-1422

Liu Z, Tyo KE, Martínez JL, Petranovic D, Nielsen J (2012) Different expression systems for production of recombinant proteins in Saccharomyces cerevisiae. *Biotechnology and Bioengineering* **109:** 1259-1268

Nayak T, Szewczyk E, Oakley CE, Osmani A, Ukil L, Murray SL, Hynes MJ, Osmani SA, Oakley BR (2006) A versatile and efficient gene-targeting system for *Aspergillus nidulans*. *Genetics* **172:** 1557-1566

Vongsangnak W, Hansen K, Nielsen J (2011) Integrated analysis of the global transcriptional response to α-amylase over-production by *Aspergillus oryzae*. *Biotechnology and Bioengineering* **108:** 1130-1139

# Primers used in this study

TAKA_amy_fw: 5’- CCACAGAAGGGATCCATGGTCGCGTGGTGGTCTCTATT

TAKA_amy_re: 5’- cctctagatctcgagatactctccacccttcacgagctact

Bar_fw: 5’- CCGCAAGGAATGGTGATGACCATGATTACGCCAAGCTAGC

Bar_re: 5’- tttagatggagcgcgaccgcatcaggcgatatctagagg
